# Theoretical and experimental study of interaction of macroheterocyclic compounds with ORF3a of SARS-CoV-2

**DOI:** 10.1038/s41598-021-99072-8

**Published:** 2021-09-30

**Authors:** Natalia Sh. Lebedeva, Yury A. Gubarev, Galina M. Mamardashvili, Svetlana V. Zaitceva, Sergey A. Zdanovich, Alena S. Malyasova, Julia V. Romanenko, Mikhail O. Koifman, Oskar I. Koifman

**Affiliations:** 1 G.A. Krestov Institute of Solution Chemistry of the Russian Academy of Sciences, 153045 Ivanovo, Russia; 2 Ivanovo State University of Chemistry and Technology, 153000 Ivanovo, Russia

**Keywords:** Drug development, Protein function predictions, Computational models, Fluorescence spectroscopy, Spectrophotometry

## Abstract

The pandemic infectious disease (Covid-19) caused by the coronavirus (SARS-CoV2) is spreading rapidly around the world. Covid-19 does an irreparable harm to the health and life of people. It also has a negative financial impact on the economies of most countries of the world. In this regard, the issue of creating drugs aimed at combating this disease is especially acute. In this work, molecular docking was used to study the docking of 23 compounds with QRF3a SARS-CoV2. The performed in silico modeling made it possible to identify leading compounds capable of exerting a potential inhibitory and virucidal effect. The leading compounds include chlorin (a drug used in PDT), iron(III)protoporphyrin (endogenous porphyrin), and tetraanthraquinone porphyrazine (an exogenous substance). Having taken into consideration the localization of ligands in the QRF3a SARS-CoV2, we have made an assumption about their influence on the pathogenesis of Covid-19. The interaction of chlorin, iron(III)protoporphyrin and protoporphyrin with the viral protein ORF3a were studied by fluorescence and UV–Vis spectroscopy. The obtained experimental results confirm the data of molecular docking. The results showed that a viral protein binds to endogenous porphyrins and chlorins, moreover, chlorin is a competitive ligand for endogenous porphyrins. Chlorin should be considered as a promising drug for repurposing.

## Introduction

Covid-19, caused by the SARS-CoV-2 coronavirus, spread rapidly around the world within a few weeks. According to the WHO report, in March 2021 there were more than 124 million confirmed cases worldwide, with more than 2 million deaths from COVID-19. So far, many COVID-19 treatment protocols have been tested, but, as a rule, they all turn out to be ineffective. The situation is complicated by the fact that there are still no approved specially formulated drugs aimed specifically at combating COVID-19.

Therefore, doctors use the existing arsenal of antiviral agents and traditional approaches to treatment. Numerous studies have shown that the SARS-CoV-2 virus contains single-stranded RNA and 28 specific proteins grouped into 16 non-structural proteins (Nsp1 to Nsp16), four structural proteins (E, M, N, and S) and eight accessory proteins (ORF3a, ORF6, ORF7a, ORF7b, ORF8, ORF9b, ORF9c and ORF10)^1–3^.

It should be noted that the problem of single-stranded viruses inactivation is a non-trivial task that has not yet been solved in the overwhelming majority of cases^4,5^. The high mutation rate of RNA viruses, combined with short generation time and large population sizes, allows the virus to develop rapidly and adapt to the host environment. This causes difficulties in the development of vaccines and antiviral drugs.

Drugs and treatment protocols that provide not only an inhibitory, but also a virucidal effect are considered to be the most promising. This is precisely the effect of macroheterocyclic tetrapyrrole compounds (MHCs) which are capable of generating singlet oxygen or other active radicals under the action of light, thereby oxidizing RNA or virus proteins.

The development of a method for the treatment of coronavirus diseases, using photoinactivation is promising, since pathogens do not have the ability to adapt to drugs, because the latter act indirectly through photo-oxidation processes. However, to ensure the efficiency of this process, MHCs must a priori not only have a high quantum yield of reactive oxygen species, but also interact with the target without forming π-π or other specific complexes affecting the aromatic system of the macroring.

Otherwise, a significant part of the energy will be dissipated, and the quantum yield of reactive oxygen species will be reduced. Bioinformatics methods, which are successfully used in order to identify leading compounds, can predict the possible variants of MHC docking with the target^6^. Another equally significant issue is the choice of target. Current therapeutic strategies against SARS-CoV-2 are aimed at obvious targets that directly determine the life cycle of the virus, namely, at the viral protease (Mpro/3CLpro)^7^, RNA polymerase^8^, S-protein. Viral protease and polymerase are involved in the viral replication stage, and the S-protein binds to host cells through angiotensin, converting enzyme 2 and transmembrane protease. It also mediates the fusion of the virus and host cell membranes^9–11^.

The spike protein is the most visible surface component of the virus and is also the main target. It is the focus of several ongoing efforts to develop a vaccine against SARS-CoV-2^12,13^. We think that targeting a drug to a single target, due to the high probability of its variability, is not an entirely correct strategy. It is more promising, in our opinion, to distinguish the leading compounds capable of attacking several targets of the virus, which are responsible for the life cycle of the virus or affect the host's immune response.

Accessory proteins encoded by the coronavirus play a crucial role in virus-host interactions and in modulating host immune responses, thereby contributing to the pathogenicity of the corona virus by means of a variety of strategies. Among them, ORF3a occupies a special position. The ORF3a protein is the largest (274 amino-acids) accessory protein in SARS-CoV-2. The expression of ORF3a in infected cells is relatively high, and ORF3a antibodies are found in significant amounts in diseased people^14,15^. Studies^16,17^ have reported that an accessory ORF3a protein encoded by SARS-CoV and SARS-CoV-2 can induce apoptosis in cells.

Apoptosis is the predominant type of programmed cell death and is recognized as an important host antiviral defense mechanism. It controls viral infection and regulates the inflammatory response. Inflammation can play both antiviral and proviral roles during the course of the disease. On the one hand, it is a part of an innate antiviral response that limits viral replication and infection. On the other hand, it promotes the spread of the virus by releasing a large number of virions. In addition, infiltration of myeloid cells at the site of inflammation leads to the spread of viral infection to dendritic cells and macrophages, which carry the virus to other sites in vivo^18^.

ORF3a SARS-CoV-2 accessory protein also affects another mechanism of host cell death – autophagy^19^. Autophagy is one of the main defense mechanisms of the cell against pathogens. ORF3a inhibits the fusion of autophagosomes and lysosomes^20,21^ due to strong interaction with the VPS39 component of the HOPS complex. This interaction negatively affects the assembly of the HOPS complex and the formation of another STX17-SNAP29-VAMP8 SNARE complex^19^, which is required for the fusion of autophagosomes and lysosomes. This ability to disrupt the fusion stage of autophagy is unique to SARS-CoV-2 ORF3a, as it has been found that the very similar SARS-CoV ORF3a cannot interact with the HOPS complex, and therefore does not affect autophagy. According to the authors^18^, it is this feature that explains the difference in pathogenicity and infectivity of these two genetically similar viruses: SARS-CoV and SARS-CoV-2.

The rest of the functional properties of ORF3a are similar for all coronaviruses^22^. Analysis of the ORF3a-deficient recombinant virus showed that ORF3a is not essential for in vitro and in vivo replication of SARS-CoV-2, but ORF3a makes a decisive contribution to viral pathogenesis^23^. Expression of ORF3a induces the activation of NF-κB (NF-kappaB is a transcription factor that controls the expression of genes for the immune response, apoptosis and the cell cycle), as well as it causes production of chemokines, fragmentation of the Golgi apparatus, stress of the endoplasmic reticulum, accumulation of intracellular vesicles and cell death^24–26^. ORF3a is a viroporin, its activity as an ion channel is necessary due to its pro-apoptotic properties. ORF3a interacts with caveolin. It suppresses type I IFN signaling, but increases the secretion of fibrinogen, which contributes to the cytokine storm^27^.

Based on the above information, it can be concluded that ORF3a SARS-CoV2 is a promising target for the treatment of COVID-19 and other coronavirus diseases, possibly due to the correction of the body's immune response and the adequate functioning of the mechanisms of autophagy and apoptosis.

## Materials and methods

### Molecular docking

**Figure 1:**
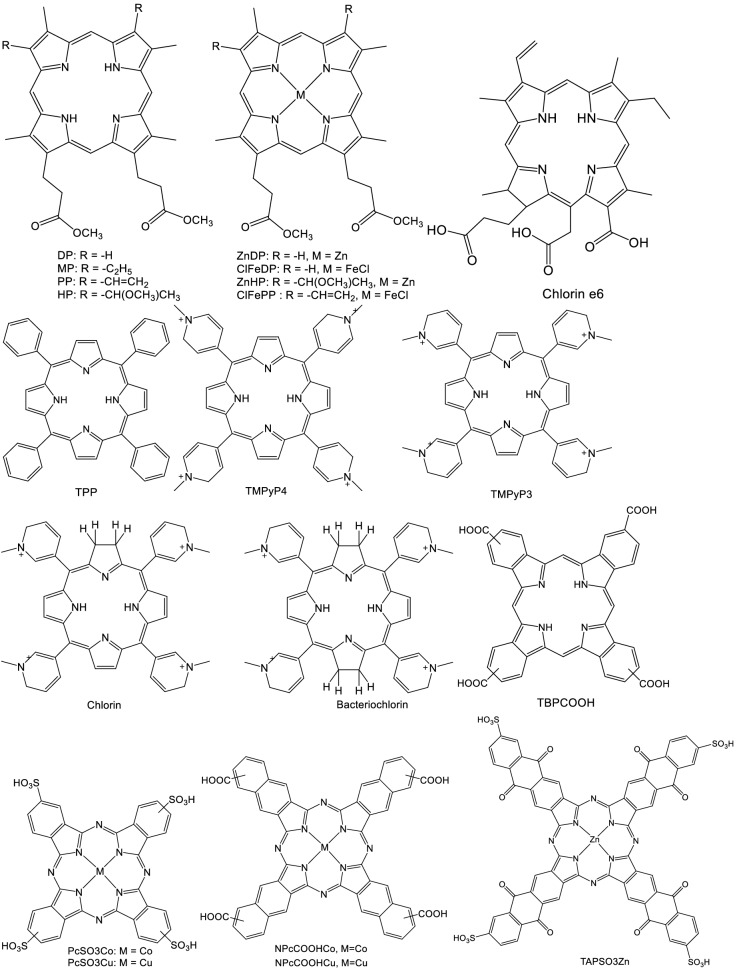
Schemes of macroheterocycles.

Structure file of ORF3a 6XDC^27^ protein was downloaded from Protein Data Bank. The structures of the macroheterocyclic compounds (Fig. 1) were minimized in the ORCA 4.0 program^28^ using the DFT b3lyp method. Molecular docking of proteins with porphyrins was performed using AutoDock Vina 1.1.2^29^ and visualized with PyMol 2.4.1. The ligand and protein structure files were prepared using AutoDockTools 1.5.6. When preparing the structure of the ligand, rotating bonds were selected automatically. Polar hydrogens were added to the protein structure. The grid matrix was sized so that the protein molecule was completely overlapped. Due to the large size of the grid matrix, the exhaustiveness parameter was increased to 512^30^. Molecular docking made it possible to find the 20 most favorable structures for each porphyrin. After analyzing the results, the most optimal positions indicated in Table 1 were selected. In the case of cationic and anionic macrocycles, the potential of the protein globule was additionally calculated by the ABPS method to analyze the docking sites^31^.

**Table 1:** Molecular docking parameters of macroheterocycles with ORF3a.

MHC	N	Affinity (kcal/mol)	Binding site of ORF3a, Fig. 2			Domain*	Potential inhibiting/inactivating	MHC	N	Affinity, kcal/mol	Binding site of ORF3a, Fig. 2			Domain*	Potential inhibiting/inactivating
			C	B	A						C	B	A		
TMPyP3	1	− 8.4	x			II, III	+ + / −	HP	1	− 8.6		x		IV	+ + / −
	6	− 8.3	x			II, III	+ + / −		5	− 7.8		x		III, IV	+ / +
	9	− 8.2	x			III	+ + / −		6	− 7.7		x		III, IV	+ / −
	15	− 8.1	x			III	+ + / −	PP	1	− 8.1		x		III, IV	+ + / +
TMPyP4	1	− 8.3	x			III	+ + / −		2	− 8.0		x		III, IV	+ / −
	9	− 7.9	x			III	+ / −		3	− 7.8		x		IV	+ / +
Chlorin	1	− 8.4	x			II, III	+ + / −	ZnHP	1	− 8.5		x		IV	+ + / +
	4	− 8.4	x			II, III	+ + / −		5	− 8.1		x		III, IV	+ + / −
	8	− 8.3	x			III	+ + / −		8	− 7.8		x		III, IV	+ / +
	11	− 8.1	x			III	+ / −	ZnDP	1	− 8.3		x		VI	+ + / −
Bacteriochlorin	1	− 8.2			x	V	+ + / −		6	− 7.7		x		III, IV	+ / −
	3	− 8.1	x			III	+ + / −		9	− 7.7		x		III, IV	+ / +
	5	− 8.1	x			III	+ + / −	NPcCOOHCo	1	− 10.6			x	V	+ + + + / −
	11	− 8.0			x	V	+ / −		5	− 10.5			x	V	+ + + + / −
TPP	1	− 8.5	x			II, III	+ + / −	NPcCOOHCu	1	− 10.7			x	V	+ + + + / −
	6	− 8.4	x			II, III	+ + / −		5	− 10.6			x	V	+ + + + / −
Chlorin e6	1	− 7.5			x	V	+ / −	PcSO3Co	1	− 9.3			x	V	+ + + / −
	2	− 7.5		x		IV	+ / −		5	− 9.1			x	V	+ + + / −
	4	− 7.4			x	V	+ / −	PcSO3Cu	1	− 9.3			x	V	+ + + / −
	7	− 7.1		x		III, IV	+ / −		5	− 9.1			x	V	+ + + / +
	9	− 7.0		x		III, IV	+ / −	NPcSO3Co	1	− 9.3			x	V	+ + + / +
ClFeDP	1	− 8.5		x		IV	+ + / −		5	− 9.1			x	V	+ + + / +
	9	− 7.9		x		III, IV	+ / +		11	− 8.7		x		III	+ + / −
	10	− 7.9		x		III, IV	+ / −		13	− 8.7		x		III	+ + / −
ClFePP	1	− 8.4		x		III, IV	+ + / −	TAPSO3Zn	1	− 11.0			x	V	+ + + + + / +
	2	− 8.2		x		IV	+ + / −		5	− 10.9			x	V	+ + + + + / +
	6	− 8.0		x		III, IV	+ / −	TBPCOOH	6	− 8.9			x	V	+ + + / +
DP	1	− 8.4		x		IV	+ + / −		12	− 8.7			x	V	+ + / −
	6	− 8.0		x		III, IV	+ / −	TPPSO3	1	− 8.5			x	V	+ + / +
	8	− 7.8		x		III, IV	+ / +		3	− 8.5			x	V	+ + / +
MP	1	− 8.0		x		III, IV	+ / +								
	2	− 8.0		x		IV	+ / −								
	6	− 7.8		x		III, IV	+ / +								

*The domain structure is indicated in accordance with the reference^22^.

### Obtaining plasmid vectors for the expression of ORF3a in E. coli cells

Using the PrimerSelect program (DNASTAR Lasergene), oligonucleotide primers carrying the NdeI and XhoI restriction sites (CTCTCATATGGATTTGTTTATGAGAATCTTCACAATTGG) were selected for PCR amplification of the genes of the SARS-CoV-2 ORF3a non-structural proteins. Plasmids pGBW-m4046959 (AddGene #145,722, respectively) carrying the sequences of the target proteins were used as a template for PCR. PCR amplification was performed _using_ Phusion® high precision DNA polymerase (New England Biolabs). One reverse primer (Uni_R (GATACTCGAGTTAATGATGGTGATGATGGTGGCTTTG)) was used. As a result, a DNA fragment of the base pairs (bp) with a length of 884 carrying the sequence of the ORF3a genes was obtained. The resulting DNA fragments were isolated from the gel, using the Cleanup S-cap kit (Evrogen).

The isolated DNA fragment having restriction sites in the terminal regions was treated with restriction endonucleases NdeI and XhoI (Thermo). The original expression vector pET22b (+) (Novagen) was treated with the same restriction endonucleases. Restriction products 870 bp in length (insert of ORF3a) were separated on an agarose gel _and_ isolated using the Cleanup S-cap kit. The isolated fragments (an insert + vector) were ligated to each other using T4 DNA ligase. The ligation mixture was used to transform competent cells of E. coli strain TOP10. The cells were plated on a solid selective (ampicillin) nutrient medium. The presence of target inserts in the clones was determined by PCR with primers ORF 3a_F/Uni_R, (Fig. 1S). In the case of plasmid pET-ORF3a, the amplicon size corresponded to the size of the target insert.

**Figure 2:**
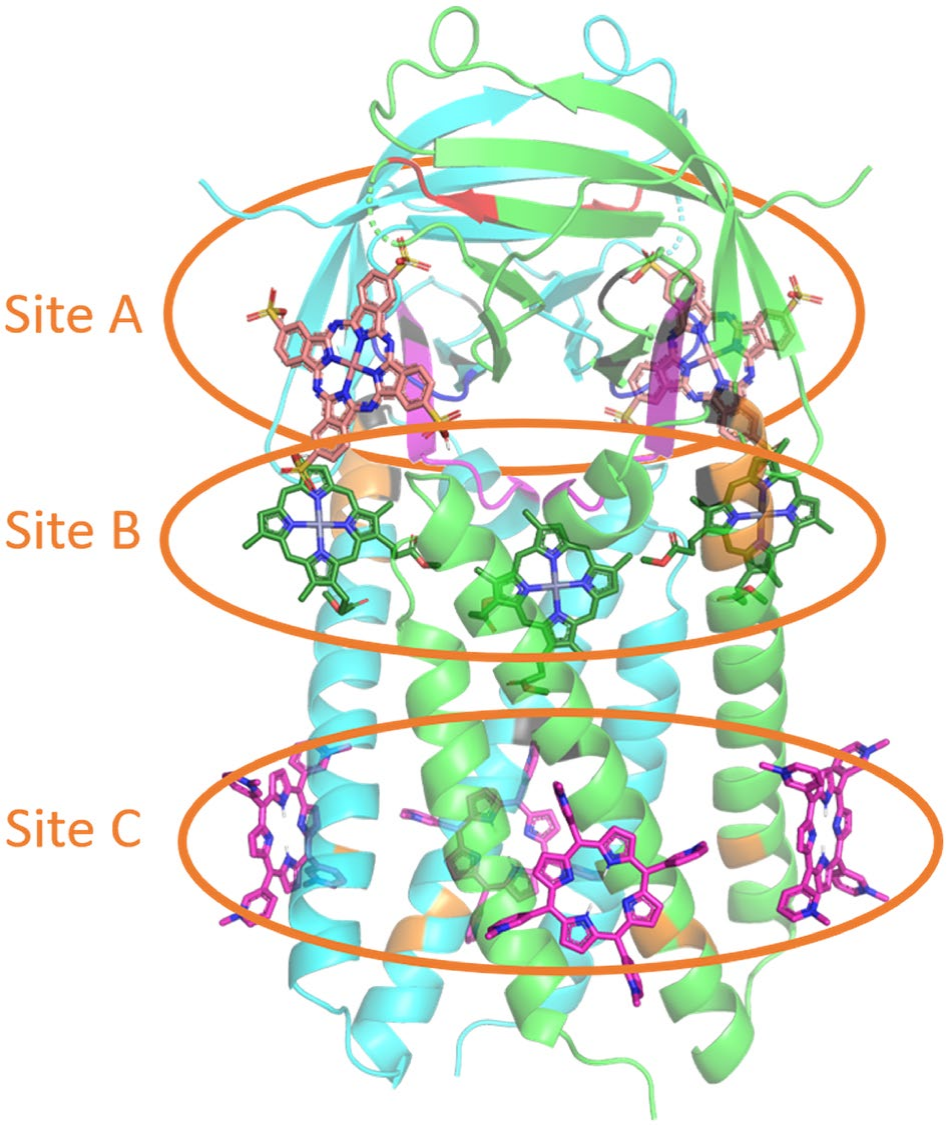
The complexes of MHCs with dimer of ORF3a. TMPyP3—magenta, ZNDP—green, NPcSO3Cu—pink. Domain structure in accordance with^22^ painted in colors: domain III—orange, domain IV—violet, domain V—dark blue, domain VI—red.

Clones containing the target inserts were grown in liquid selective nutrient medium; plasmid DNA was _isolated_ using the Plasmid Miniprep kit (Evrogen). Additionally, the presence of target inserts in the final constructs was confirmed by restriction analysis (Fig. 2S). After treatment with restriction endonuclease XhoI, the plasmids became linear. After treatment with restriction endonucleases NdeI and XhoI, the plasmids were cleaved into fragments containing the sequences of the vector (5364 bp) and ORF3a inserts—870 bp.

The obtained expression constructions of pET-ORF3a were used at the next stage of the study to obtain producers of ORF3a proteins.

### Preparation of the recombinant ORF3a protein

The E. coli BL21 (DE3) strain was used to express the target proteins. It is a derivative of the BL21 strain _and_ contains an artificial cassette in the λ prophage encoding RNA polymerase of the T7 phage under the control of an inducible lac promoter. Transformants of this strain with pET-ORF3a plasmids were obtained by chemical transformation.

Transformants were cultured in 2YT medium at 37 °C with vigorous stirring (220 rpm) in an Innova 40 incubator shaker (New Brunswick) until an optical density of A550 = 0.5 OU was achieved. The optical density of the culture was monitored, using a DEN-1 photometer-_densitometer_ (Biosan). The lac promoter of the RNA polymerase gene of the T7 phage of the host strain was induced by adding IPTG to a final concentration of 0.5 mM. Then the bacteria were cultivated with vigorous stirring for 12 h at a temperature of 28 °C, the cells were precipitated by centrifugation. The cells were resuspended in PBS-R buffer (20 mM Na_2_HPO_4_, 100 mM NaCl, pH 7.4) and disrupted by ultrasound at a frequency of 22 kHz sequentially 30 times for 10 s with intermediate cooling of the transmitter and suspension in an ice bath for 10 s using ultrasonic disintegrator Labsonic P (Sartorius). To the supernatant containing the fraction of soluble proteins, NaCl was added to a concentration of 500 mM, then a 10% polyethyleneimine solution was added dropwise with stirring to a concentration of 0.1% to precipitate nucleic acids. The lysate was centrifuged (10 min, 15,000 g at 4 °C), the supernatant was saved.

To the supernatant containing the fraction of soluble proteins, NaCl was added to a concentration of 500 mM, then a 10% polyethyleneimine solution was added dropwise with stirring to a concentration of 0.1% to precipitate nucleic acids. The lysate was centrifuged (10 min, 15,000 g at 4 °C), the supernatant was saved. Imidazole was added to the supernatant to a concentration of 30 mM, the pH was adjusted with a hydrochloric acid solution to 7.4. The resulting solution was filtered through a syringe filter _with_ a pore diameter of 0.22 μm.

Purification of the target proteins from the cell lysate was carried out by liquid chromatography using the AKTA pure 25 M1 system (GE Healthcare). At the C-terminus of the amino _acid_ sequences of the recombinant ORF3a protein, there is an oligohistidine "tail" (6 histidine residues), which makes it possible to purify these proteins using metal-affinity chromatography.

**Figure 3:**
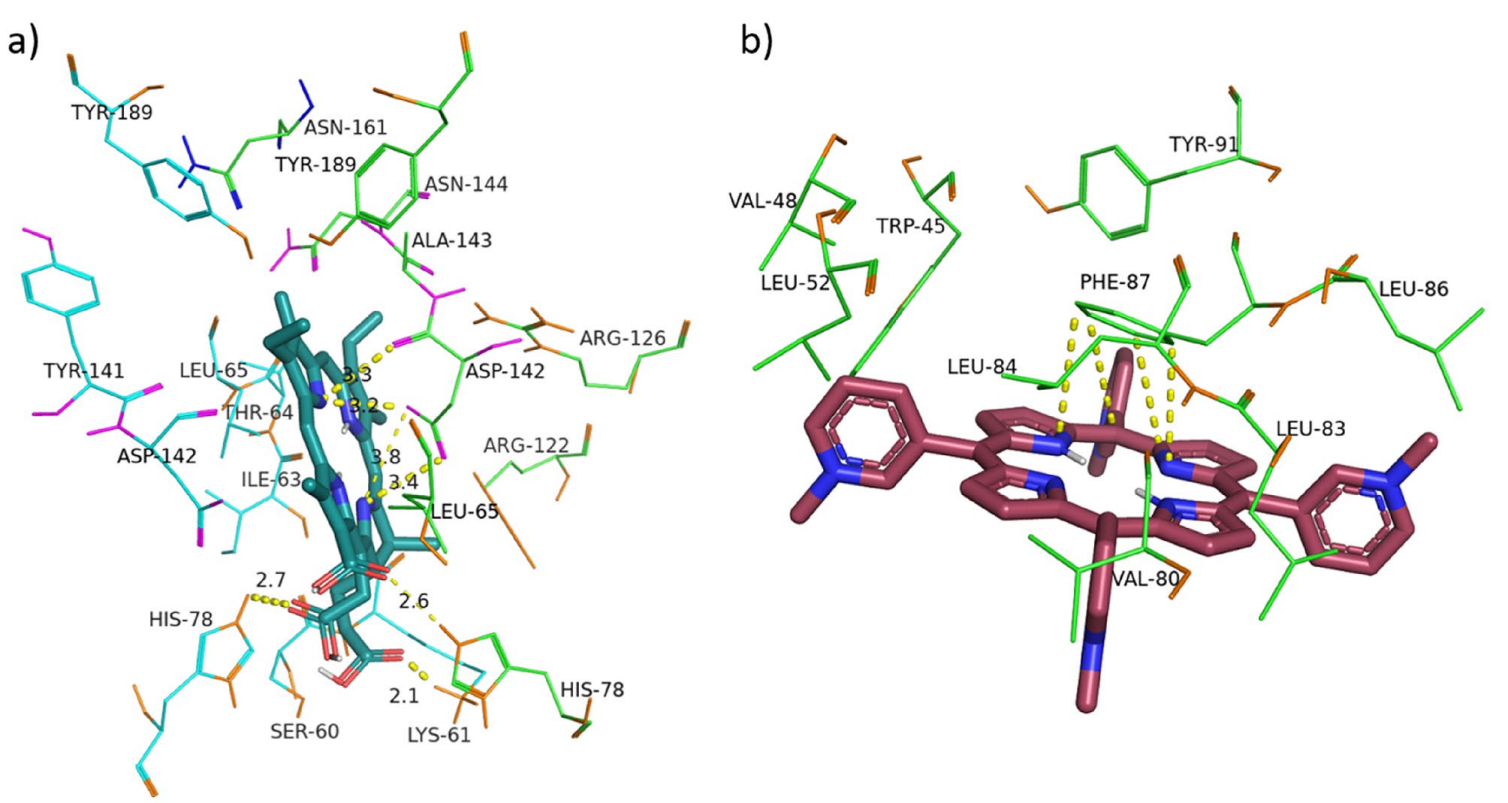
The complexes of Chlorin e6, complex 2 (**a**) and Chlorin, complex 1 (**b**) with dimer of ORF3a, chain A—green, chain B—blue.

The cell lysate was loaded onto a HisTrap FF 5 ml column (Cytiva) equilibrated with PBS-B buffer (20 mM Na_2_HPO_4_, 500 mM NaCl, 30 mM imidazole, pH 7.4) at a flow rate of 5 ml / min. The protein immobilized on the column was washed with 50 ml of PBS-B buffer at a flow rate of 5 ml / min. The target protein was eluted with 20 ml of PBS-E buffer (20 mM Na_2_HPO_4_, 500 mM NaCl, 500 mM imidazole, pH 7.4) at a flow rate of 5 ml / min. Protein-containing fractions were collected in 2 ml test tubes, guiding by the readings of the UV detector of the chromatographic system (Fig. 3S).

The obtained fractions were analyzed by electrophoresis in 12% PAGE. Bands at the level of 32 kDa were visible in the gel, and this value corresponded to the calculated masses of the target ORF3a protein. The purity of the obtained proteins was analyzed using the GelAnalyzer2010a program (Lazar software). The purity of the samples in all cycles of production and extraction was 90–93%. Protein solutions were concentrated, using Amicon Ultra-4 centrifugal filters (Merck) to a volume of 1 ml.

Concentrated ORF3a protein was transferred into PBS buffer (Merck) pH 7.4, using PD 25 Midi columns (GE Healthcare). Protein concentration was determined by the bicinchoninic acid method using the BCA Protein Assay Kit (Thermo) according to the manufacturer's recommendations.

The authenticity of the obtained protein samples was determined by the method of immunoblotting with antibodies to the oligohistidine sequence present at the C-terminus of the proteins. After electrophoresis in PAGE, the proteins were transferred onto an Immobilon-P polyvinylidene fluoride membrane (Millipore). The membrane was blocked for 1 h with 5% milk powder in TBS-T buffer (50 mM Tris, 500 mM NaCl, 0.05% Tween-20, pH 7.0). The membrane was incubated for 1 h in a 1:1500 solution of primary antibodies (mouse 6x-His Epitope Tag Antibody, Thermo) in 5% dry milk in TBS-T buffer. The membrane was washed three times with TBS-T buffer for 5 min. The membrane was incubated for 1 h at 1:1000 in a solution of secondary antibodies (HRP Goat Anti-Mouse Ig, BDbiosciences) in 5% dry milk in TBS-T buffer. The membrane was washed as described above. The membrane was incubated for 5 min in a Clarity Western ECL Substrate peroxidase substrate solution (Bio-Rad) was visualized, using the ChemiDoc system (Bio-Rad). In all cases, a specific colour of the bands of the target protein was observed on the membrane.

The work on the expression and purification of ORF3a proteins was carried out by the Nizhny Novgorod National Research University after N. I. Lobachevsky within the framework of contract No. 33 dated 05/13/2021.

### Synthesis of macrocycles

Protoporphyrin IX d.m.e., iron (III) protoporphyrin IX d.m.e. were purchased in Sigma-Aldrich. Chlorin was synthesized in accordance to well-known and proven method^32^. All compounds were isolated and purified, and their structure was confirmed.

### Spectral study

UV–Vis and fluorescence spectra were registered using an AvaSpec-2048 spectrophotometer (Avantes BV, Netherlands), with a temperature-controlled cell at 25 °C in 10 mm quartz cuvettes. The monochromatic LED with spectral maximum at 525 nm (B5B-433-B525, Roitner Lasertechnik Gmbh., Austria) was used as excitation light source in the fluorescence study. All measurements were performed in the PBS. PP and ClFePP were preliminarily dissolved in DMF and then added to protein solution in PBS.

## Results and discussion

The aim of this work is to describe molecular modeling of the ORF3a protein docking with a number of macroheterocyclic compounds in order to identify the leading compounds capable of inhibiting or inactivating, in particular ORF3a, and SARS-CoV2 in general. Compounds of the porphyrin, chlorin, porphyriazine, and phthalocyanine series (Fig. 1) were chosen as MHCs. They differ in the degree of aromaticity, hydrophobic / hydrophilic character, as well as in the presence / absence of peripheral substituents and their symmetry.

Most of the ORF3a coronaviruses proteins function in a dimeric form, about 10% of these proteins are tetramers^27,33^, therefore, the MHC was docked to the ORF3a SARS-CoV-2 dimer. The results obtained are shown in Table 1, 1S. Judging by the data obtained, the SARS-CoV-2 ORF3a dimer has 3 main MHC binding sites (Fig. 2). Cationic porphyrins, chlorin, bacteriochlorin, and tetraphenylporphyrin are primarily localized in the lower part of ORF3a (Fig. 2). Nature porphyrins and their metal complexes with iron (III) and zinc (II) ions form complexes with the dimer, binding to the protein in the upper part of the helices (Fig. 2). Anionic porphyrins, phthalocyanines, porphyrazines bind to the cytosolic site ORF3a SARS-CoV-2 (Fig. 2).

The effect of ORF3a on cellular functions is not well understood, but there is reliable information that ORF3a is a class IIIa viroporin with the N-terminus oriented to the extracellular space and with the C-terminus located on the cytosolic side of the membrane^18^. The activity of the ORF3a ion channel is essential for its pro-apoptotic properties and plays an important role in virus release^34^. According to the literature^27^, the helical part of the protein, located in the lipid layer of the membrane, forms and regulates the flow of cations. In this regard, taking into account the results of molecular docking presented in Table 1, it can be assumed that chlorin E6, nature porphyrins, and NPcSO3Co will inhibit both the process of incorporation of ORF3a into the cell membrane and the activity of ion channels. Judging by the obtained energies of interaction of MHC with ORF3a, the nature porphyrins and their metal complexes will have the best inhibitory ability, since they provide the maximum binding energy (− 8.3 to − 8.5 kcal/mol).

The binding energy of chlorin E6 with ORF3a is lower and amounts to − 7.5 kcal/mol. In addition, as shown by the results of molecular docking, chlorin E6 demonstrates several variants of docking with ORF3a, but in all cases, chlorin E6 forms either π-π-complexes with aromatic amino-acid residues of the protein, or H-bonds with the participation of the porphyrin reaction center. Consequently, chlorin E6 cannot act as an effective inhibitor due to the low binding energy with ORF3a, and also as a photoinactivator, because of the formation of specific complexes the energy of the excited triplet state will dissipate to a greater extent. This dissipation, in turn, will lead to a decrease in the quantum release of active oxygen species. Nature porphyrins and their metal complexes exhibit the ability to form several complexes with ORF3a with similar energies (Table 1). In some cases, they form specific donor–acceptor, π-π or hydrogen bonds with the amino-acid residues of the polypeptide chain, affecting the π-system of the MHC (Table 1S). However, among the most probable variants of MHC docking with ORF3a complexes were found those in which the aromatic system is not involved. Therefore, these porphyrins (HP, MP, PP and ZnHP) can be potential photoinactivators.

The mechanism of the effect of SARS-CoV-2 ORF3a on cellular compartments is being investigated, and is likely to be studied for many more years but the current situation requires answers as soon as possible. Therefore, to our mind, the approach outlined in the work^22^ is very productive. The authors investigated the presence of nonsynonymous mutations in the ORF3a protein in SARS-CoV2 compared to SARS-CoV and other coronaviruses. Due to the presence of conserved regions and the known pathogenesis for preexisting coronaviruses, in the case of ORF3a SARS-CoV-2, it was possible to find the domains and establish their role in the host cell. In total, the authors^22^ have identified 6 domains. We compared the identified binding sites of macroheterocyclic compounds by the ORF3a protein with the domains found in the work^22^ (Table 1).

Of all the studied MHCs, only TPP, chlorin, and TMPyP3 are located in the immediate vicinity of domain II (Table 1). The binding energy of these compounds with ORF3a is comparable and lies in the range of 8.4–8.5 kcal / mol. Domain II (Residues 36–40) of ORF3a has been reported^22^ to bind TRAF through the TRAF-3 binding motif (TRAF proteins bind and mediate signaling from members of the TNFR superfamily. This protein is involved in signaling CD40—a member of the TNFR family important for activation immune response). In addition, this domain activates NF-kB and the NLRP3 inflammasome. Thus, the interaction of MHCs with domain II or fragments of the ORF3a amino-acid chain located close to domain II will make it possible to inhibit the processes of ORF3a fusion with host proteins that affect the immune response. It should be noted that complexes of MHCs with the amino-acid sequence near domain II of ORF3a are energetically more favourable from all possible variants of MHCs docking with ORF3a (Table 1). In this case, TPP, chlorin and TMPyP3 are involved in the π-π-interaction between the aromatic system of MHC and Phe87 ORF3a, and this phenomenon will reduce the photoactivity of MHC. Taking into account the solubility, quantum yields of reactive oxygen species, as well as the availability of approvals for using in medical practice, chlorin should be considered the undoubted leader.

Domain III (Residues 91, 93, 109, 127–133) is responsible for the formation of cation channels in the ORF3a dimer. This domain is spatially separated and comprises two regions located in the upper and lower parts of the spirals (Fig. 3). The leading compounds "working" at the top of the spirals were discussed above. With regard to the potential for inhibiting or inactivating the activity of ORF3a ion channels in the lower part of the helices, only TMPyP3 (complexes 1, 6, 9, 15), TMPyP4 (complexes 1, 9), chlorin (complexes 1, 4, 8, 11), TPP (complexes 1,6), and NPcSO3Co (complexes 11, 13) can reveal inhibiting ability. Photoinactivation is unlikely, since the listed MHCs form π-π complexes with Phe87, Tyr113, Phe114, Trp69 (Table 1S, Fig. 3).

Domain IV (Residues 141–149) contains a caveolin-binding motif, provides uptake and delivery of the ORF3a protein into plasma, endomembranes, and the Golgi apparatus. Chlorin E6, nature porphyrins and their metal complexes bind to this domain (Table 1). The energetic stability of MHCs complexes with the amino- acid sequence of domain IV ORF3a decreases in the following order of MHCs: ClFePP (complexes 1, 9) < PP (complexes 1, 2) ≤ ZnHP (complexes 5, 8) ≤ MP (complexes 1, 6) ≤ DP (complexes 6, 8) HP (complexes 5, 6) ≤ ZnDP (complexes 6, 9) < chlorin E6 (complexes 7, 9) (Table 1). It should be noted that nature porphyrins and their metal complexes have fairly close binding energies (Table 1), which significantly exceed this characteristic for the ORF3a complex with chlorin E6. The reason for this phenomenon is the formation for each porphyrin of several hydrogen bonds between 6.7 peripheral substituents of the macroring and amino acid residues (Table 1S). It is noteworthy that the introduction of oxygen-containing substituents into the 2, 4 positions of the macroring in the case of hematoporphyrin and its metal complex actually does not affect the energy of binding to the protein. ClFePP should be recognized as a leading compound potentially capable of exhibiting the highest inhibitory ability towards domain IV. On the contrary, ClFeDP (complex 9), DP (complex 8), HP (complex 5), MP (complex 6), ZnHP (complex 8), and ZnDP (complex 9) can act as potential photoinactivators, which do not form specific complexes affecting the aromatic system of the macroring.

Domain V (Residues 160–163) is a conservative motif that plays a role in the transport of the ORF3a protein from the Golgi apparatus to the cell and intracellular membranes, lipid droplets, and lysosomes. Domain V is responsible for the intracellular transport of ORF3a. Bacteriochlorin (complexes 1, 11), chlorin E6 (complexes 1, 4), as well as all the studied anionic porphinins, phthalocyaninesand tetraanthraquinone porphyrazines dock with the domain V of SARS-COV2 ORF3a. Judging by the energy stability of the corresponding complexes, the inhibitory ability of MHC decreases in the following order: TAPSO3Zn < NPcCOOHCu < NPcCOOHCo < NPcSO3 Co < PcSO3Co = PcSO3Cu = NPcSO3Co < TBPCOOH < TPPSO3 < Bacteriochlorin (complex 1) < chlorin E6 (complex 1). The expansion of the aromatic system on passing from chlorin compounds to tetraphenylporphyrin, tetrabenzoporphyrin, phthalocyanines, naphthalocyanines and further to tetraanthraquinone porphyrazines leads to a significant increase in the binding energy of MHCs with the domain V of ORF3a SARS-CoV2. In this case, several leading compounds can be distinguished which do not form specific complexes with the participation of the aromatic system of the macroring and can act as potentially effective photoinactivators. These include TAPSO3Zn (complexes 1, 5), NPcSO3Co (complexes 1, 5), PcSO3Cu (complex 5), TBPCOOH (complexes 6, 12), TPPSO3 (complexes 1, 3).

The next stage of the work was an experimental study of the complexing properties of the ORF3a protein in relation to exogenous porphyrins, as well as to endogenous chlorin, which, judging by the results of molecular docking, binds to ORF3a and can be an inhibitor and an inactivator of the viral protein. As shown above, according to the calculations, there are more promising leading compounds in comparison with chlorin, but chlorin has one clear advantage: it is the main drug in the medicine for photodynamic therapy. It can be considered as an exclusive drug for drug repurposing.

**Figure 4:**
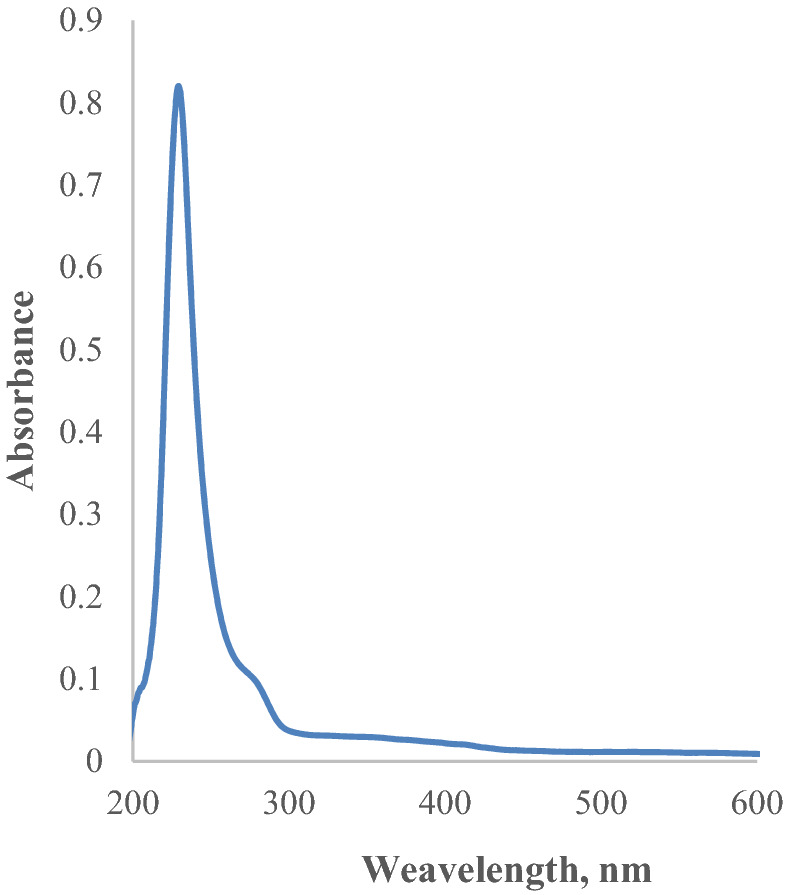
UV–Vis spectrum of ORF3a in PBS at pH = 7.4.

The ORF3a protein was expressed, and spectral information about the ORF3a protein was obtained. The maximum absorption of ORF3a is at a wavelength of 230 nm, absorption is asymmetric with a shoulder of about 278 nm (Fig. 4). We suppose that the absorption occurs due to ORF3a dimeric structures in the region of long wavelengths.

**Figure 5:**
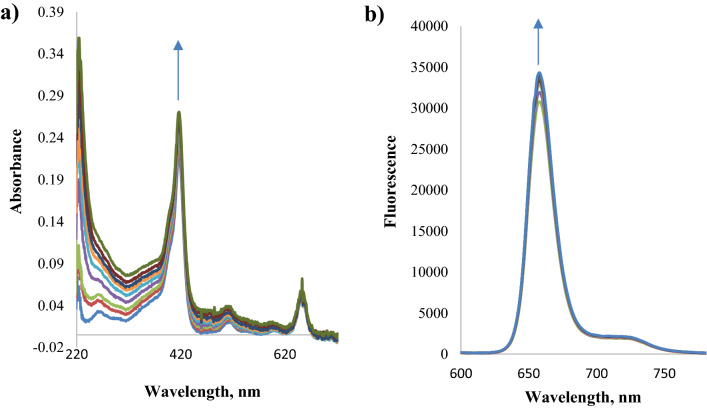
Direct titration of chlorin (1·10^−6^ M) by ORF3a protein (1·10^−7^ ÷ 1·10^−6^ M) in PBS: (**a**) UV–Vis spectra (**b**) fluorescence spectra.

**Figure 6:**
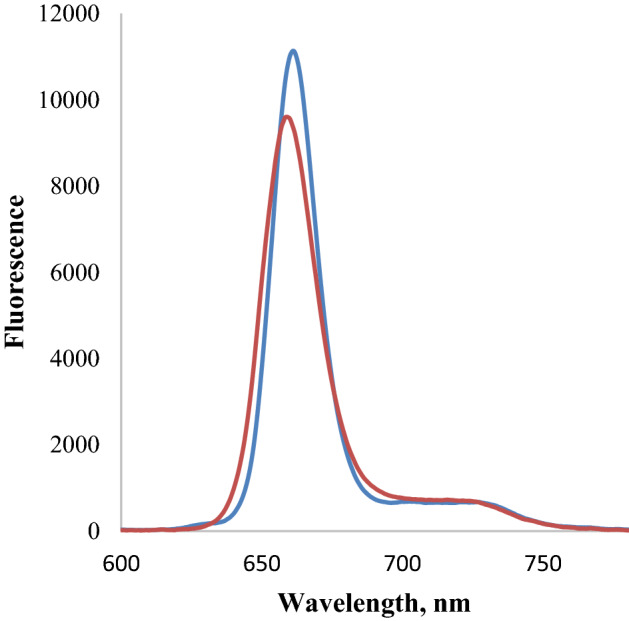
Chlorin fluorescence spectra in DMF (blue line) and PBS (orange line).

To study the interaction of ORF3a with chlorin, direct and back titration was carried out. During titration of chlorin with protein (back titration), an increase in optical density was recorded in the region of the Soret band, and its decrease in the region of 650 nm was revealed. In this case, the fluorescence of chlorin increased (Fig. 5). Chlorin fluoresces more intensely in a non-polar medium (Fig. 6), therefore, an increase in fluorescence in the complex with the ORF3a protein confirmed the results of molecular docking of ORF3a to chlorin, according to which chlorin binds to the hydrophobic region of the protein—site C (Fig. 2, Table 1S). All of the above spectral changes indicate the formation of a complex of ORF3a with chlorin. It should be noted that in all the systems under study, a significant increase in reflection was recorded. Probably, the binding of ORF3a to chlorin causes protein aggregation. The Scatchard parameters calculated from the spectral data are given in Table 2. In the case of protein titration with chlorin (direct titration), the absorption of individual chlorin was taken into account. According to the results of titration of the ORF3a protein with chlorin, the following conclusions can be drawn: the difference absorption spectra with an increase in the amount of chlorin and, depending on the analyzed spectral range, do not change symbatically. In the region of 655 nm the absorption decreases, in the region of 483 nm it does not increase monotonically. Difference spectra are recorded with isobestic points.

**Table 2:** The affinity and number of binding sites for ORF3a and its complexes with porphyrins in relation to chlorin according to the results of direct and reverse titration.

Complex	Scatchard constant	Number of binding sites	Type of titration
ORF3a * Chlorin	1.04 × 10^6^	1.1	UV–Vis, reverse
	1.07 × 10^6^	0.5	Fluorescence, reverse
	7.1 × 10^5^	0.9	UV–Vis, direct
	4.6 × 10^4^	1.7	Fluorescence, direct
ORF3a*PP	7.9 × 10^4^	1.0	UV–Vis, direct
	7.5 × 10^4^	2.0	Fluorescence, direct
ORF3a*ClFePP	4.68 × 10^5^	2.5	UV–Vis, direct
	8.3 × 10^4^	2.7	Fluorescence, direct

Judging by the difference fluorescence spectra, the fluorescence of chlorin in the complex with the ORF3a protein is higher than in the solvent. In direct titration, as in the case of back titration, it was found that with an increase in the amount of chlorin in the system under study, the reflection increased, but to a much lesser extent. This is quite unexpected since the concentration of ORF3a was significantly higher in direct titration. Probably, the presence of reflection did not allow us to correctly estimate the parameters of the complexation of ORF3a with chlorin from the data of electronic absorption spectroscopy; to some extent, this contribution was taken into account in the procedure for correcting the fluorescence spectra. Therefore, the affinity constants calculated from fluorescence data are more reliable (Table 2).

**Figure 7:**
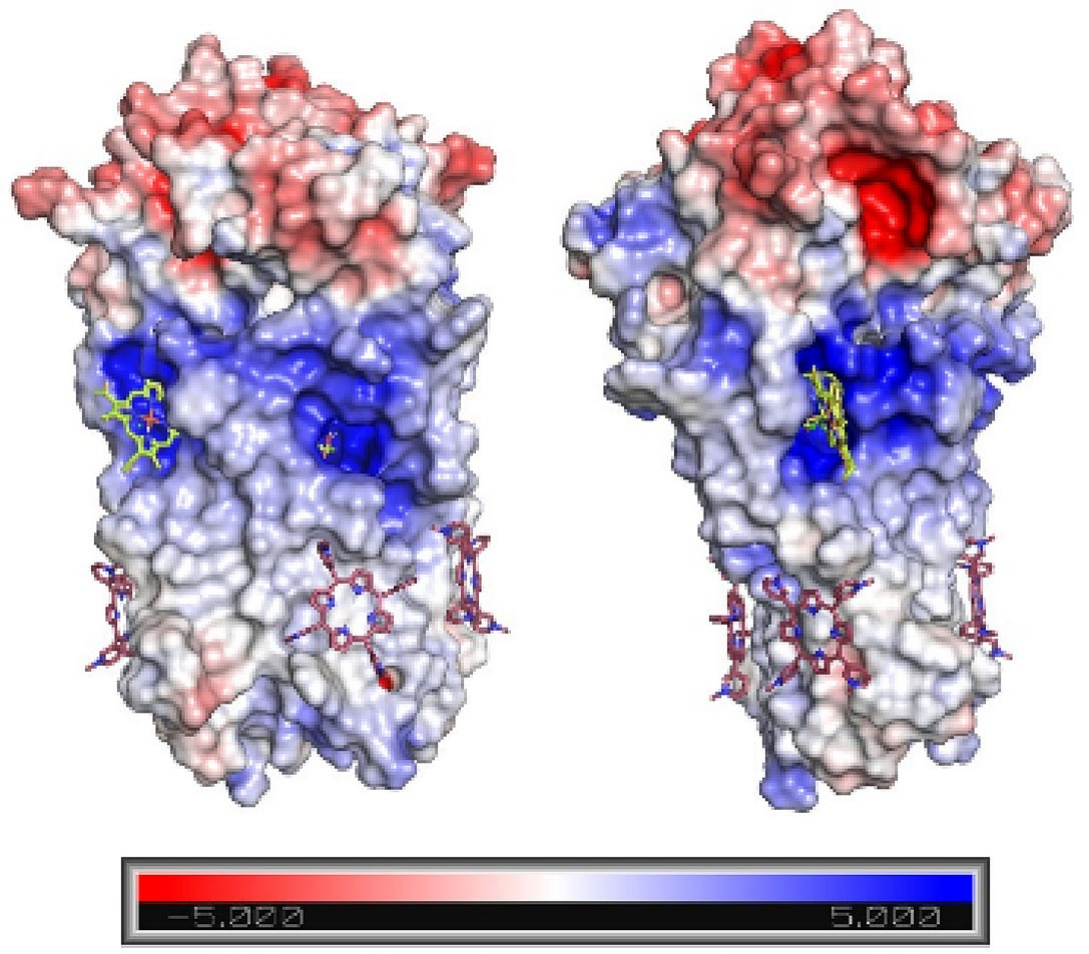
Structures of ORF3a complexes with ClFePP (green) and Chlorin (pink) according to the results of molecular docking.

Chlorin exhibited a completely different behaviour upon binding to complexes of ORF3a with porphyrins (PP and ClFePP). Due to the hydrophobicity of porphyrin compounds and insolubility in aqueous media, ORF3a complexes with PP and ClFePP were obtained by introducing equimolar amounts of solutions of porphyrins in DMF into protein solutions. In this case, the DMF concentration did not exceed 1 × 10^–4^ M. The formation of ORF3a complexes with porphyrins was confirmed by the aggregation stability of solutions. On the contrary, when analogous amounts of solutions of porphyrins in DMF were added to a pure buffer, precipitation of porphyrins was observed almost immediately. As noted above, the binding of chlorin to a protein differed from its binding to complexes of a protein with porphyrins. The main difference was the decrease in chlorin fluorescence upon binding to the ORF3a*PP and ORF3a*ClFePP complexes. Judging by the results, the molecular docking of porphyrins (protoporphyrin, iron (III) chloride, protoporphyrin) and chlorin with ORF3a occurs at different sites (Fig. 7). The obtained affinity constants of the complexes ORF3a*PP and ORF3a*ClFePP for chlorin were almost 2 times higher than the affinity of ORF3a for chlorin. Probably, protein binding of porphyrins causes reorganization of site C (Fig. 2) and promotes chlorin binding; cooperative binding is implemented. It is likely that this reorganization of site C leads to a change in the nature of the amino acid environment in the ORF3a-porphyrin-chlorin complexes. Judging by the decrease in chlorin fluorescence, the amino acid environment of the MHC becomes more hydrophilic. As with the protein, when the ORF3a*PP and ORF3a*ClFePP complexes were titrated with chlorin, an increase in the reflection was observed in the spectra, i.e. binding to chlorin leads to protein aggregation, which is most pronounced for ORF3a*ClFePP.

## Conclusions

Thus, molecular docking made it possible to find leading compounds among macroheterocycles, potentially capable of inhibiting and photoinactivating various functions of ORF3a. Chlorin is the most promising among macroheterocycles capable of inhibiting ORF3a domain II, which affects the immune response. ClFePP is the most promising in its group of inactivators of the ORF3a ion channel activity. It is advisable to experimentally investigate HP, MP, PP, and ZnHP as photoinactivators. Tetraanthraquinone porphyriazines and naphthalocyanines should be considered effective inhibitors of intracellular transport of the ORF3a protein.

Experimental studies of the interaction of the viral protein ORF3a with exogenous porphyrins and chlorin, a drug used for PDT, have been carried out. The results showed that a viral protein that affects the host's immune response binds to endogenous porphyrins and chlorins. Chlorin should be considered as a promising drug for repurposing; further in vitro studies are needed.

## Supplementary Information

Supplementary Information.
