# Supplementary material for: Theoretical and experimental study of interaction of macroheterocyclic compounds with ORF3a of SARS-CoV-2
Source: Sci Rep. 2021 Sep 30;11:19481. doi: 10.1038/s41598-021-99072-8 (PMC8484456; doi:10.1038/s41598-021-99072-8)
Supplement: Supplementary file 1 — Supplementary Information. [file 41598_2021_99072_MOESM1_ESM.docx]

Theoretical and experimental study of interaction of macroheterocyclic compounds with ORF3a of SARS-CoV-2

Natalia Sh. Lebedeva^1^, Yury A. Gubarev^1^, Galina M. Mamardashvili^1^, Svetlana V. Zaitceva^1^, Sergey A. Zdanovich^1^, Alena S. Malyasova^2^, Julia V. Romanenko^2^, Mikhail O. Koifman^1,2^, Oskar I. Koifman^1,2^

^1^ G.A. Krestov Institute of Solution Chemistry of the Russian academy of Sciences, 153045, Ivanovo, Russia

^2^ Ivanovo State University of Chemistry and Technology, 153000, Ivanovo, Russia

**Table 1S.** Molecular docking parameters of macroheterocycles with ORF3a

| **MHC** | **N** | **Specific interactions between MHC and ORF3a residues. Res/length, Å/place, M-binds to macroring** | | **The residues of ORF3a near 4Å of MHC** |
| --- | --- | --- | --- | --- |
|  |  | **H-bonds** | **π-π bonds** |  |
| TMPyP3 | 1 |  | Phe87a/3.6/M | B:45,48,52,80,83,84,86,87,91 |
|  | 6 |  | Phe87b/3.6/M | A:45,48,54,80,83,84,86,87,91 |
|  | 9 |  | Tyr113b/3.6/M  Phe114b/3.6 | B:90,106,110,113,114,116,117 |
|  | 15 |  | Tyr113a/3.6/M  Phe114a/3.6 | A:93,106,110,113,114,116,117 |
| TMPyP4 | 1 |  | Tyr113a/3.6/M  Phe114a/3.6 | A:93,106,110,113,114,117 |
|  | 9 |  | Tyr113b/3.6/M  Phe114b/3.6 | B:90,106,113,114,117,120 |
| Chlorin | 1 |  | Phe87a/3.6/M | B:45,48,52,80,83,84,86,87,91 |
|  | 4 |  | Phe87b/3.6/M | A:45,48,54,80,83,84,86,87,91 |
|  | 8 |  | Tyr113b/3.6/M  Phe114b/3.6 | B:90,106,110,113,114,116,117 |
|  | 11 |  | Tyr113a/3.6/M  Phe114a/3.6 | A:93,106,110,113,114,116,117 |
| Bacteriochlorin | 1 | Ser162/3.3/M  Val163/3.3M |  | A:141,172,173,183,185,188,190  B:145,161-165,235, 237 |
|  | 3 |  | Tyr113a/3.5/M Phe114a/3.5 | A:93,106,110,113,114,117 |
|  | 5 |  | Tyr113b/3.5/M  Phe114b/3.8 | B:90,93,106,110,113,114,117,120 |
|  | 11 | Val163/3.3/M  Thr190/4.0 |  | A:145,161-165,235,237  B:66,67,141,172,173,183,188,190 |
| TPP | 1 |  | Phe87b/3.5/M | B:45,48,52,80,83,84,87,91 |
|  | 6 |  | Phe87a/3.5/M | A:45,48,52,80,83,84,87,91 |
| Chlorin e6 | 1 | Ser165/3.7/M  Asn234/2.0  Glu226/3.5  His227/2.3  Asp173/2.6  Asp183/2.5 |  | A:172,173,181,183,185,225-227  B:163-165,234,235,237 |
|  | 2 | Asp142a/3.2/3.8/3.4/3.8/M  His78a/2.6  Lys61b/2.1  His78b/2.7 |  | A:65,78,122,126,142-144,161,189  B:60,61,63-65,78,141,142,189 |
|  | 4 | Ser165a/3.7/M  Asn234a/2.0/2.5  Asp173b/2.5/2.5  His227/2.3 |  | A:163-165,234,235,237  B:172,173,181,183,185,226-228 |
|  | 7 | Thr64/3.8/M  Ile118/2.6 | Tyr206/3.8/M | A:118,121,122,125,144,205-209  B:61-64,66 |
|  | 9 | Thr64/3.1/M  Lys66/1.9/2.7  Tyr206/3.3  Phe207/2.5  Thr208/3.3 |  | A:62-65,66  B:144,145,205-209 |
| ClFeDP | 1 | Gln57a/2.7  Lys61a/2.9/3.1  His78b/2.4  Arg122b/2.7 | His78b/3.4/M | A:57,60,61,65,78,81,142  B:57,60,61,63,65,75,78,122,142 |
|  | 9 | Arg126b/2.4/2.7  Arg122b/2.0 |  | A:62,64,66  B:118,122,125,126,142,205-208 |
|  | 10 | Arg122a/2.1  Arg126a/2.5/2.6 | Tyr206a/3.8/M |  |
| ClFePP | 1 | Arg122a/2.0/2.5  Arg126a/2.3/2.4/2.8  Ala143a/2.4  Thr64b/3.8/Fe |  | A:118,122,126,129,142-144,146,204-207  B:61-65,66 |
|  | 2 | His78a/2.4  Ser60a/2.1  Gln57a/2.8  Lys61a/3.2/3.3  Arg122b/2.6  Asp42b/2.8 | His78b/3.8/M | A:62,64,66  B:118,122,125,126,142,205-208 |
|  | 6 | Ala143b/2.4  Arg126b/2/2.5/3.1  Asn144b/2.0  Tyr206b/3.0  Thr64b/3.9/Fe |  | A:118,122,125,126,142,144,205-208  B:62,64,66 |
| DP | 1 | Asp142a/2.6/2.7/3.5/M |  | A:60,65,78,122,142,144,161,189  B:61,63-65,141-143,189 |
|  | 6 | Arg126a/2.5/2.5  Tyr206a/2.3 | Tyr206a/3.6/M | A:118,122,125,126,142,205-208  B:62,64,66 |
|  | 8 | Arg122b/1.9/3.3  Arg126b/2.6/2.6 |  | A:62,64,66  B:118,122,125,126,142,144,205-208 |
| HP | 1 | Lys61a/2.8  Gln57a/3.2  Arg122b/2.1  Ser60b/2.3  Ile63b/2.3 | His78b/3.6/M | A:57,60-63,65,78,122,141,142,189  B:57,60-63,65,78,122,142 |
|  | 5 | Lys61a/2.1  Ile63a/2.1  Lys66a/2.5  Arg122b/2.6  Tyr206b/2.5 |  | A:61-64,66  B:122,126,129,143-146,205-208 |
|  | 6 | Arg122a/2.3/3.4  Asp142a/3.5/2.4  Arg126a/2.2  Ala143a/2.4  Ile63b/1.9  Thr64b/3.9/M |  | A:122,126,142-145,205-207  B:61-64,66 |
| MP | 1 | Arg122/2.2/2.4  Arg126/2.6 |  | A:122,126,142-145,205-208  B:61-64,66 |
|  | 2 | Tyr189a/2.9  Arg126a/3.4  Arg122a/2.2/2.4 | His78b/3.6/M | A:57,60,61,65,78,122,126,141-143,189,206  B:57,60,61,63,65,78,122,142,189 |
|  | 6 | Ile63a/1.9  Arg122b/2.2/2.9  Arg126b/2.2  Ala143b/2.1 |  | A:61-64,66  B:122,126,142-145,205-208 |
| PP | 1 | Arg122a/2.5  Tyr206a/2.4  Arg126a/2.2/2.3/2.5  Asn144a/2.0 |  | A:118,122,126,129,143-145,205-207  B:61-64,66 |
|  | 2 | Lys61a/3.1/3.2  Gln57a/2.7  Ser60a/2.6/2.0  Arg122b/2.6 | His78b/3.5/M |  |
|  | 3 | Arg126a/2.2/3.4  Arg122a/2.1/2.4  Asp142b/2.6  Tyr206b/2.4 |  | A:61-64,66  B:118,122,126,142-144,205-207 |
| ZnHP | 1 | Arg122a/2.3  Ile63a/2.1/2.7  Asp142b/3.0  Tyr189b/2.1/3.0  Lys61b/2.8  Gln57b/3.2 |  | A:57,60-63,65,78,122,142  B:57,60-63,65,78,122,141,142,189 |
|  | 5 | Arg126a/2.4/2.7  Tyr206a/2.8  Arg122a/3.3 | Tyr206a/3.3/M | A:122,126,129,143-146,205-208  B:62,64,66 |
|  | 8 | Arg126a/2.4/2.7  Ala143b/2.5  Asn144b/2.2  Tyr206b/2.8 |  |  |
| ZnDP | 1 | His78a/2.5  Ser60a/2.0  Gln57a/2.8  Lys61a/2.8/3.0  Arg122b/2.9 | His78b/4.0/M | A:57,60,61,65,78,81,142  B:57,60,61,63,65,75,78,121,142 |
|  | 6 | Arg122a/1.9  Arg126a/2.3/2.6 | Tyr206a/3.6/M | A:118,122,126,142,205-208  B:62,64,66 |
|  | 9 | Arg126b/2.3/2.7  Arg122b/1.9/3.2 |  | A:62,64,66  B:118,122,125,126,142,205-208 |
| NPcCOOHCo | 1 | Arg68a/2.3  Gln70a/2.4  Lys67a/2.4/M |  | A:64,66-68,70,172,173,183,185,190-192,227,228  B:162-164,210,235 |
|  | 5 | Ser165a/2.4  Lys67b/3.1/M  Gln70b/2.0  Thr64b/3.1  Thr190b/3.0  Lys192b/2.5  Asp173b/3.2 |  | A:163,165,210,235  B:64,66-68,70,172,173,183,190-192,227,228 |
| NPcCOOHCu | 1 | Arg68a/2.2  Gln70a/2.4  Ser162b/3.6/Cu  Val163b/3.0/M |  | A:64,66-68,70,172, 173,183,185,190-192,227,228  B:162-165,210,235 |
|  | 5 | Ser165a/2.4  Ser163a/3.3/M  Gln70a/2.1  Thr190b/2.5  Lys192b/2.6  Asp173b/3.1 |  | A:163,165,210,235  B:64,66-68,69,172,173,183,190-192,227,228 |
| PcSO3Co | 1 | Lys66a/2.3/2.8  Lys67a/2.9/M  Arg68a/2.5  Tyr141a/3.0  Asp183a/2.0/2.2  Glu181a/3.2  Asp210b/2.3  Lys235b/2.1/3.0 |  | A:66-68,141,181, 183,185,190  B:162-165,210,235 |
|  | 5 | Asp210a/3.3  Lys235a/2.2  Lys67b/3.2/M  Arg68b/2.7  Tyr141b/2.5  Asp183b/3.5 |  | A:161-165,210,235  B:66-68,141,181, 183, 185,190 |
| PcSO3Cu | 1 | Lys66a/2.3/2.7  Arg68a/2.6  Tyr141a/2.6  Ser162b/3.7/Cu  Lys235b/2.5/2.4  Asp210b/3.1 |  | A:66-68,141,181, 183,185,190  B:161-165,210,235 |
|  | 5 | Asp210a/3.2  Lys235a/2.3  Asp183b/1.7  Tyr141b/2.5  Arg68b/2.7  Lys66b/2.2 |  | A:161-161,210,235  B:66-68,172,181, 183,185,190 |
| NPcSO3Co | 1 | Arg68a/2.5  Gln70a/2.3  Thr64a/3.0  Thr190a/2.3  Lys67a/2.3  Ser165b/2.4 |  | A:64,66-68,70,172,173,183,185,190-192  B:162-166,210,235 |
|  | 5 | Ser165a/2.3  Thr64b/2.9  Gln70b/2.2  Lys67b/2.3  Arg68b/2.5 |  | A:163-166,210,235  B:64,66-68,70,141, 172,173,183,190-192,227,228 |
|  | 11 | Lys136b/2.3 | Trp69b/4.3 | B:56,68,69,72,73,76,77,123,127,135,136,139,154 |
|  | 13 | Tyr154a/2.9 | Trp69a/3.9 | A:56,68,69,72,73,76,77,79,127,136,139,154 |
| TAPSO3Zn | 1 | Gln70a/2.1/2.9  Arg68a/2.8  Lys192a/2.1  Thr190a/3.9  Glu226a/3.0  Ser165b/2.1 |  | A:64,66-68,70,137,183,190-192,226-228 |
|  | 5 | Ser165a/2.1  Glu226b/3.0  Lys192b/2.1  Thr190b/4.0  Arg68b/2.8  Gln70b/2.1/2.9 |  | A:165,235  B:64,66-68,70,137, 141,154,183,190-192,226-228 |
| TBPCOOH | 6 | Lys66a/2.3/2.2  Arg68a/3.3  Asp183a/2.9  Tyr141a/2.4 |  | A:66-68,141,170, 172,183,185,190  B:161-165,235 |
|  | 12 | Lys235a/2.3  Lys66b/2.4  Arg68b/2.9 |  | A:162-165,235,237  B:66-68,141,172,183,185,190 |
| TPPSO3 | 1 | Lys66a/2.1  Arg68a/2.5  Tyr141a/2.3  Gly172a/2.8 |  | A:66-68,71, 141, 172,173,181,183,185,190,228  B:145,161-164,235, 237 |
|  | 3 | Tyr141b/2.3  Arg68b/2.5  Lys66b/2.4  Gly172b/2.8  Asp183b/3.6 |  | A:145,161-164,235,237  B:66-68,71,141, 172,173,181,183,185,190,228 |


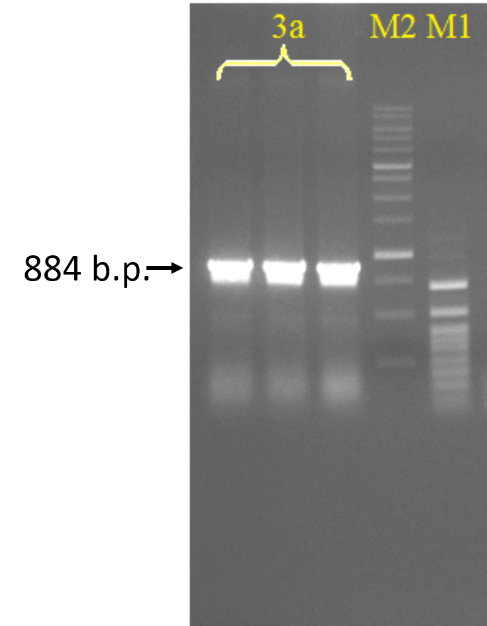


Figure 1S. Analysis of the results of PCR of clones of the plasmid pET-ORF3a. M1 and M2 are molecular weight markers of 100+ bp and 1 kb DNA Ladder (Evrogen), respectively.


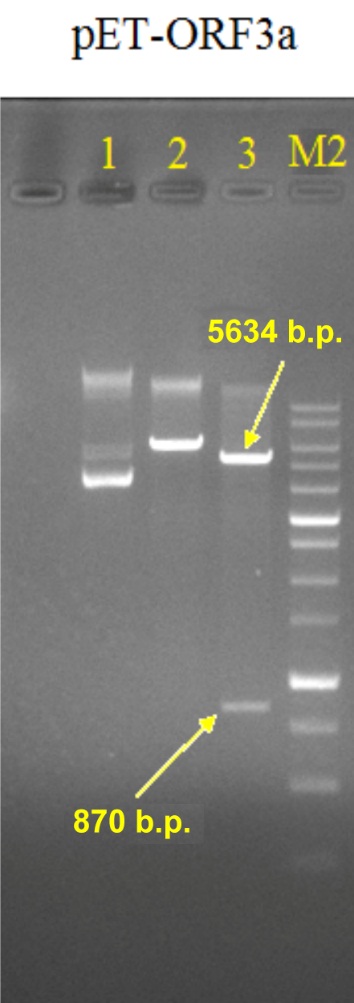


Figure 2S. Restriction analysis of plasmids pET-ORF3a. 1 - original plasmids, 2 - after treatment with restriction endonuclease XhoI, 3 - after treatment with restriction endonucleases NdeI and XhoI. M1 and M2 are molecular weight markers of 100+ bp and 1 kb DNA Ladder (Evrogen), respectively.


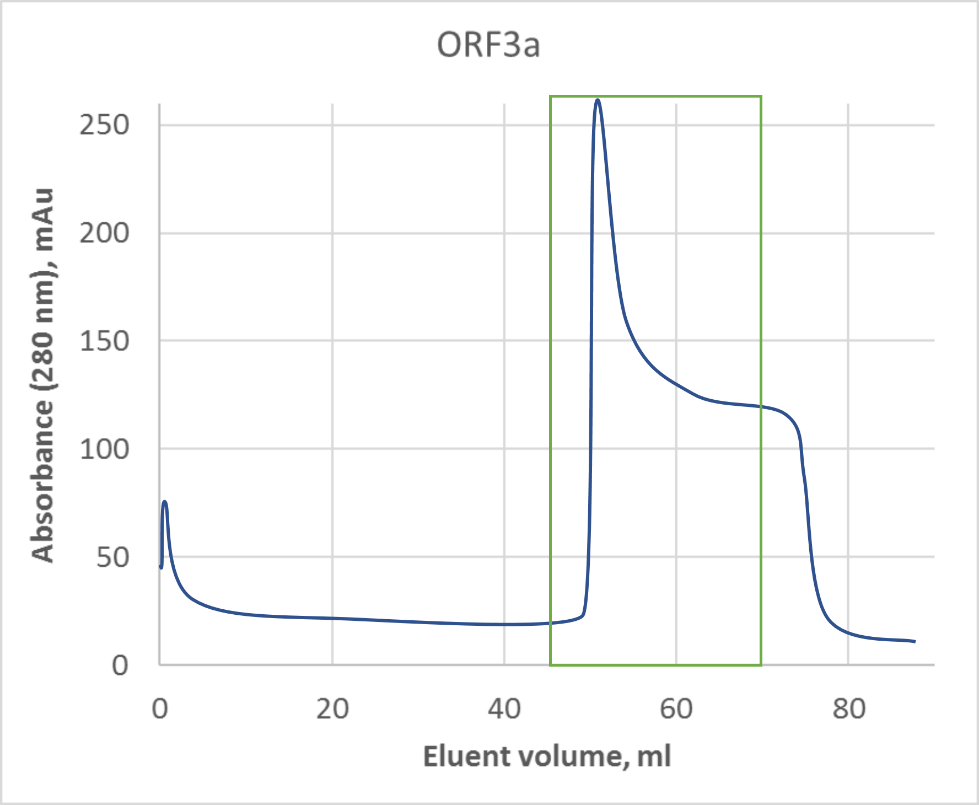


Figure 3S. Chromatograms of ORF3а, ORF8, and ORF10 proteins elution from a nickel-affinity column (Ni Sepharose sorbent). The green line shows the gradient of the elution buffer.
